# Endocannabinoid Modulation of Microglial Phenotypes in Neuropathology

**DOI:** 10.3389/fneur.2020.00087

**Published:** 2020-02-14

**Authors:** Mikiei Tanaka, Scott Sackett, Yumin Zhang

**Affiliations:** Department of Anatomy, Physiology and Genetics, Uniformed Services University Health Sciences, Bethesda, MD, United States

**Keywords:** neuroinflammation, immunomodulation, microglia subtype, alternative activation, M0/M1/M2 polarization, endocannabinoids, CB2 receptor agonist, animal disease model

## Abstract

Microglia, the resident immune cells of the central nervous system, mediate brain homeostasis by controlling neuronal proliferation/differentiation and synaptic activity. In response to external signals from neuropathological conditions, homeostatic (M0) microglia can adopt one of two activation states: the classical (M1) activation state, which secretes mediators of the proinflammatory response, and the alternative (M2) activation state, which presumably mediates the resolution of neuroinflammation and tissue repair/remodeling. Since chronic inflammatory activation of microglia is correlated with several neurodegenerative diseases, functional modulation of microglial phenotypes has been considered as a potential therapeutic strategy. The endocannabinoid (eCB) system, composed of cannabinoid receptors and ligands and their metabolic/biosynthetic enzymes, has been shown to activate anti-inflammatory signaling pathways that modulate immune cell functions. Growing evidence has demonstrated that endogenous, synthetic, and plant-derived eCB agonists possess therapeutic effects on several neuropathologies; however, the molecular mechanisms that mediate the anti-inflammatory effects have not yet been identified. Over the last decade, it has been revealed that the eCB system modulates microglial activation and population. In this review, we thoroughly examine recent studies on microglial phenotype modulation by eCB in neuroinflammatory and neurodegenerative disease conditions. We hypothesize that cannabinoid 2 receptor (CB2R) signaling shifts the balance of expression between neuroinflammatory (M1-type) genes, neuroprotective (M2-type) genes, and homeostatic (M0-type) genes toward the latter two gene expressions, by which microglia acquire therapeutic functionality.

## Introduction

In the last several decades, a growing body of evidence has revealed an intricate cross talk between neurons and immune cells to maintain brain homeostasis (1, 2). If this delicate equilibrium is disrupted by any pathological stimuli, the inflammatory response can be exaggerated in the central nervous system (CNS). In response to neuroinflammation, microglia, the resident macrophages of the CNS, undergo morphological, phenotypic, and functional changes. Evidence has shown that upon activation microglia can cause deleterious effects on neuronal cells by releasing reactive oxygen and nitrogen species, cytokines, chemokines, and other inflammatory mediators. The dying neurons, in turn, release more stimulatory factors, which exaggerate the activation of microglia. This vicious cycle contributes to the pathogenesis of neurodegenerative diseases. On the other hand, several recent studies have shown that under certain experimental settings microglia, similar to macrophages in the periphery, display an alternative activation state that presumably leads to cytoprotective effects by secreting trophic factors and tissue remodeling molecules. Moreover, microglia *in vivo* have been observed to display characteristics that resemble the alternative activation state, which is designated as the M2 state as opposed to the classical activation M1 state.

Microglia/macrophages in the alternative activation state are believed to be critically involved in neuronal cell repair, tissue remodeling, including debris clearance, and the resolution of inflammation (3). Thus, in order to halt the vicious cycle of neuroinflammation and prevent neuronal injury, it is crucial to control or modulate microglial activation states rather than eliminate microglial activity (4, 5). Over the past decade, the neuroprotective effects of endocannabinoids (eCB) have received a significant amount of attention. Numerous studies have shown that activation of eCB signaling can suppress microglial activation and ameliorate neurodegeneration in several neurological diseases. The therapeutic mechanisms of eCB signaling are at least partially due to the modulation of microglial polarization. In this review, we summarize recent studies, mainly published in the last decade, regarding the regulation of microglial polarization by the eCB system in both *in vitro* cell cultures and disease animal models. We propose that cannabinoid type 2 receptor (CB2R)-mediated signaling plays a vital role in the modulation of microglial polarization, and we evaluate some issues that should be addressed. Although we briefly outline the eCB system in the CNS and microglial activation hereafter, several excellent and comprehensive review articles regarding the eCB system (6–9) and microglial/macrophage polarization (10–13) are available; readers are encouraged to review these articles to understand the related topics.

## Key Pharmacological eCB Components in the CNS

The cannabinoid type 1 receptor (CB1R) was first cloned as the binding receptor for Δ^9^-tetrahydrocannabinol, the main psychologically active compound in *Cannabis sativa* (14), and CB2R was later cloned in 1993 (15). Since then, a variety of plant-derived and synthetic compounds that target cannabinoid (CB) receptors have been identified and developed as agonists or antagonists. In parallel, endogenous CB ligands were also discovered; anandamide (AEA), which was discovered in 1992 (16), and 2-arachidonoyl glycerol (2-AG), discovered in 1995 (17, 18), are the best-characterized eCB ligands. AEA binds to both CB receptors as a partial agonist, while 2-AG binds to these receptors as a full agonist (19–21). Later on, several new components of the eCB system, including ethanolamine, glycerol, or amino acid derivatives of acyl fatty acids, such as N-palmitoylethanolamine, 2-oleoylglycerol, and N-arachidonoylglycine, were identified in the CNS and shown to be involved in eCB signaling.

CB1R is one of the most abundantly expressed G-protein coupled receptors in the CNS and is primarily expressed in neurons. CB1R is localized in presynaptic terminals where its activation negatively modulates neurotransmission. Thus, CB1R signaling is the critical neuronal regulator for the control of motor function, emotion, cognition, memory, and analgesia (22). CB2R is highly expressed in immune cells, such as B cells, NK cells, and macrophages, in the peripheral nervous system (PNS) and predominantly in microglia in the CNS. Moreover, since CB2R expression is upregulated in tissues under pathological stimuli (23), CB2R is regarded as the central component of the eCB system involving the inflammatory response. With regard to downstream signaling, both CB1R and CB2R have two independent pathways: the canonical G-protein-dependent pathway and the non-canonical G-protein-independent pathway. Upon ligand binding, adenylyl cyclase is inhibited by the activation of G_i/o_, the main G protein subunit associated with CBs. As a result, cAMP is reduced, followed by modulation of its downstream signal transducers, such as protein kinase A. CBs are also associated with G_βγ_ proteins, which initiate other signaling pathways that activate certain calcium and potassium ion channels and several mitogen-activated protein kinases (MAPKs), such as extracellular signal-regulated protein kinase (ERK), c-Jun NH2 terminal kinase (JNK), and p38 MAPK pathways (24). These pathways are involved in cell proliferation, migration, and cytokine production. Additionally, non-canonical CB signaling can be mediated by β-arrestin (25). β-arrestin is the scaffold protein associated with CBs, and it regulates their internalization and desensitization. β-arrestin and several other signal molecules are recruited to form complexes with CB receptors to act as either receptor signal transducers or terminators (26). Thus, CBs mediate multiple signaling pathways that intricately cross talk with each other. The output signaling is impacted by surrounding microenvironments and intracellular conditions. In addition, the selectivity and preference of downstream CB signaling is determined by the CB ligands, endogenous AEA and 2-AG, or the synthetic CB agonists (27, 28). Considering these CB ligands may also have off-target effects (29), regulation of this complex signaling system by eCB modulation has not yet been completely elucidated.

**Figure 1:**
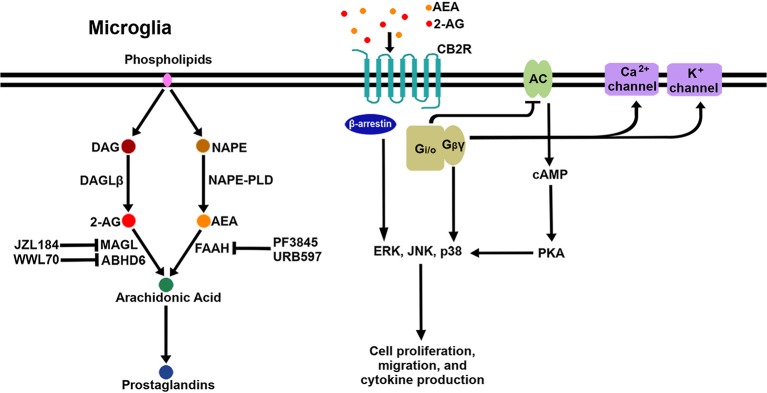
Schematic signaling pathways and biosynthesis/degradation of endocannabinoids in microglia. When eCB bind to CB2R on the microglial cell surface, the receptor initiates signaling through the canonical G-protein-dependent pathway and the non-canonical G-protein-independent pathway. Adenylyl cyclase (AC) is inhibited by the activation of G_i/o_ proteins; as a result, cAMP is reduced, followed by modulation of PKA signaling. G_βγ_ proteins activate certain calcium and potassium ion channels. Additionally, β-arrestin is recruited to CB2R to act as a receptor signal transducer or terminator. Three arms of the CB2R signaling pathway activate multiple downstream pathways, including several MAPKs (ERK, JNK, and p38 MAPK). AEA and 2-AG are mainly biosynthesized from NAPE by NAPE-PLD and from DAG by DAGLβ, respectively. AEA is degraded to arachidonic acid by FAAH, which is inhibited by PF3845 and URB597. 2-AG is degraded to arachidonic acid by MAGL and ABHD6, which are inhibited by JZL184 and WWL70, respectively. Arachidonic acid is a key precursor for prostaglandins.

After the discovery of the endogenous ligands, several enzymes responsible for their biosynthesis and metabolism in the CNS were identified. The major synthesizing enzymes for 2-AG are diacylglycerol lipase (DAGL)α and DAGLβ (30), by which diacylglycerol is converted to 2-AG. DAGLα is the major biosynthesizing enzyme in neurons, while DAGLβ is the major biosynthesizing enzyme in microglia (31). There are multiple pathways responsible for the biosynthesis of AEA; N-acyl phosphatidylethanolamine phospholipase D (NAPE-PLD), which catalyzes the cleavage of N-acylethanolamine from N-arachidonoyl-phosphatidylethanolamine, is considered the main biosynthetic enzyme (32). 2-AG degradation occurs mainly through monoacylglycerol lipase (MAGL) (33, 34) but also through α-β-hydrolase domain (ABHD)6 (35) and ABHD12 (36) to a lesser extent (37). The chief degrading enzyme of AEA is fatty acid amide hydrolase (FAAH). In addition, eicosanoid biosynthetic enzymes such as cyclooxygeanase-2 (COX-2), lipoxygenase12/15, and cytochrome P450 (CYP450) are also involved in eCB metabolism due to the structural similarity between eCB ligands and the eicosanoid precursor, arachidonic acid (38). In order to boost eCB signaling, several enzyme inhibitors have been developed to block the activity of the eCB-degrading enzymes. The inhibitors of MAGL (i.e., JZL184), ABHD6 (i.e., WWL70), and FAAH (i.e., PF3845, URB597) have been extensively investigated with regard to their pharmacological efficacy *in vitro* and in *in vivo* disease models. Figure 1 shows a schematic diagram of CB2R signaling pathways and eCB metabolic pathways, including enzyme inhibitors, in microglia.

As new eCB ligands were discovered, additional receptors were identified and coupled with eCB signaling and function. These receptors include transient receptor potential vanilloid 1 (TRPV1) (39), GPR55 (40), GPR18 (41), and peroxisome proliferator-activated receptors (PPARs) (42). PPARγ is activated when it binds to one of several lipid mediators, such as AEA and other N-acylethanolamines; it then acts as a transcription factor for a subset of genes that are involved in energy and lipid metabolism, oxidative stress inhibition, and the anti-inflammatory response (43). Furthermore, PPARγ has been recognized as a modulator of microglial alternative activation since treatment with a PPARγ agonist triggers alternative activation of microglia *in vitro* and in a chronic stress model (44).

## Function Of Microglia Under Activated Conditions

Although there is still debate about macrophage/microglial ontogeny, microglia are currently believed to develop from early erythromyeloid progenitor cells that originate in the yolk sac and migrate to the CNS, whereas monocyte-derived macrophages develop from hematopoietic stem cells (45). Microglia have unique physiological functions in the CNS (46), including synaptic organization (47), trophic support for neurons (48), and regulation of neuronal excitability (49). Nevertheless, macrophages and microglia share many functions as sentinels and effectors of the immune response in the PNS and CNS, respectively. Upon brain injury, a substantial number of blood macrophages are activated and infiltrate the parenchyma. Since the immune response of infiltrated macrophages is quite similar to that of microglia, the immunological roles of microglia and macrophages are difficult to distinguish; nevertheless, these two cell types can generally be identified by the expression levels of cell surface marker CD45 (CD45^low^ for microglia; CD45^high^ for macrophage) or by specific markers for microglia, such as Tmem119 (50) and P2ry12 (51). Therefore, most of the experimental data for the immune response and phenotype characterization described in this review are thought to be influenced by both types of cells unless specified.

### Initiation of the Microglial Immune Response

Regulation of microglial activation is mostly dependent on the interaction of microglia with molecules in the brain parenchyma. These extracellular molecules secreted from adjacent cells are recognized by a variety of different receptors expressed in the cytoplasmic membrane or cytoplasm; these receptors are known as Pattern Recognition Receptors (PRRs). The PRRs expressed in glial cells mainly consist of Toll-like receptors (TLRs), NOD-like receptors (NLRs), and scavenger receptors (SRs) (52). Each type of PRR binds to specific molecules, some of which are known as Pathogen Associated Molecular Patterns (PAMPs). PAMPs are molecules of exogenous origin and are associated with pathogens; PAMPs include bacterial membrane components, such as lipoprotein or peptidoglycan, and bacterial nucleic acid (unmethylated DNA or RNA) (53). On the other hand, certain types of PRRs can react with Danger Associated Molecular Patterns (DAMPs), which are of intracellular origin and are released to the extracellular space or other compartments after CNS injury (54). DAMPs include a variety of cellular components, such as proteins (Amyloid β, S100, heat shock proteins, thioredoxin, high-mobility group box 1), nucleic acids (mitochondrial DNA/RNA), and molecules from the extracellular matrix (hyaluronic acid, fibronectin) (55). In addition, small molecules like ATP and calcium ions can drive microglia to move toward the lesion site and trigger phenotypic change (56, 57). Considerable data show that PRRs are essential for surveillance of CNS homeostasis and are among the first responders to CNS injury. Both PAMPs and DAMPs directly induce proinflammatory cascades and the formation of the inflammasome, and therefore they mediate the release of cytokines (58). Microglial activation is, in turn, regulated by the cytokines or chemokines released from the immune cells at lesion sites in a paracrine and/or autocrine manner.

### Microglial Classical Activation

Under physiological conditions, microglia maintain a ramified cell shape. However, in response to abnormal microenvironments and factors, microglia adopt a phagocytic phenotype, in which the small soma becomes enlarged, and the number and length of processes progressively decrease until the cell attains an amoeboid morphology (55, 59–61). As a first line of defense, the classical activation (M1) of microglia is geared toward killing pathogens or infected cells, and it subsequently triggers the antigen presentation response to induce the adaptive immune system. Reactive oxygen or nitrogen species are a powerful tool for destroying pathogens and infected cells. These molecular species are mainly derived from inducible nitric oxide synthase (iNOS), myeloperoxidase, and NADPH oxidase in reactive microglia. During classical activation, these enzymes are upregulated and activated, and, as a result, the production of reactive oxygen or nitrogen species is increased. Regarding the adaptive immune response, several of the associated receptors and enzymes are upregulated. For instance, major histocompatibility complex II (MHCII), CD86, and Fcγ receptors are upregulated in the classical activation state. These receptors are involved in the antigen-presenting activity of microglia and interact with T cells that have infiltrated the brain parenchyma (62).

### Microglial Alternative Activation

**Figure 2:**
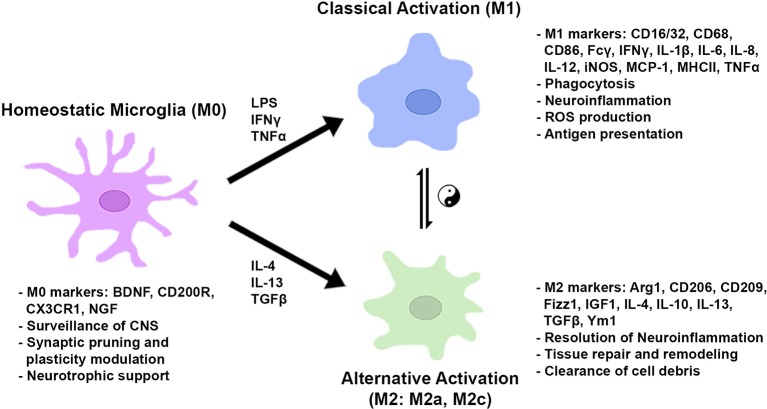
Microglial metabolic and gene regulatory states based on *in vitro* studies. In normal conditions, microglia take on a homeostatic state expressing genes for CNS surveillance, synaptic modulation, and neuronal trophic support. In the presence of pro-inflammatory stimuli, microglia are classically activated to induce genes for phagocytosis, ROS production, and antigen presentation. Anti-inflammatory cytokines activate genes to resolve inflammation, repair/remodel tissue, and clear cell debris. Microglial cells can shift between gene regulatory states dependent on environmental cues and stimulatory conditions.

After the onset of classical activation to eliminate pathogens, resolution of inflammation and restoration of brain homeostasis are required. The initial classical activation is followed by a secondary alternative activation (M2), which is important for wound healing and suppression of inflammation. The existence of two distinct phenotypes was first theorized based on the original finding that the IL-4-mediated inflammatory response adopts an alternative activation associated with a reduction in proinflammatory cytokines in macrophages (63). Subsequent studies demonstrated that the alternative (M2) phenotype was characterized by the augmented expression of anti-inflammatory cytokines (i.e., IL-4, IL-10, and IL-13), trophic factors, such as transforming growth factor-β (TGF-β) and insulin-like growth factor 1 (IGF1), and metabolic or tissue remodeling genes, such as arginase1 (Arg1) and chitinase-3-like protein 3 (Ym1) (64–69). Arg1 catalyzes L-arginine to urea and L-ornithine, which is the precursor for polyamine biosynthesis and inhibits classical activation (M1) by competing with NO generation from iNOS. Furthermore, the alternative (M2) phenotype was found to be further classified into multiple subtypes based on different sets of cytokine expression and receptor profiles, similar to macrophages. Treatment with IL-4 and IL-13 induced the expression of SRs for phagocytosis and anti-inflammatory molecules, such as Ym1, Fizz1, and IGF1. The subtype induced by these cytokines, classified as M2a, is presumably important for the resolution of inflammation and the clearance of cell debris. The M2c subtype, which is induced by TGF-β, IL-10, and glucocorticoids *in vitro*, is characterized by a deactivating phenotype and postulated to be involved in tissue remodeling and matrix deposition (69). In macrophages there exists another alternative state, M2b, which is more closely related to the M1 phenotype; however, the M2b activation state is not clearly seen in microglia. Of note, current classification of microglia/macrophages into certain phenotypes is based on cell culture studies *in vitro*. The microglia/macrophages are stimulated by individual cytokines, such as IFNγ, IL-4, or TGF-β, and a subset of genes and the cell morphology are observed (70). Figure 2 shows a schematic diagram of the microglial phenotypes and their typical gene markers.

However, in brain pathologies and even in physiological milieu (e.g. aging) (71), the mixture of cytokines, the variety of the surrounding matrix, and the different microenvironmental factors influence the polarization and gene expression of microglia/macrophages (12). In several studies using animal models, microglia and macrophage phenotypes have not been clearly defined and often have mixed profiles due to the environmental milieu in which both classical and alternative activation inducing cues are present (72). Therefore, the two phenotypes or markers of both phenotypes are sometimes observed simultaneously in the same cell (73). The typical phenotypes of the M1 and M2 states have not been proven *in vivo*. Some transcriptomic studies at the single-cell level have attempted to characterize microglial heterogeneity in disease animal models or pathological conditions (74–77); however, the studies have not identified microglial subset characteristics for the M1 or M2 polarized phenotypes. It was proposed that the M1/M2 phenotype is an oversimplification or even non-existent *in vivo* based on experimental evidence (78, 79). However, the terms M1 and M2 do not only indicate specific microglial subsets; they also indicate individual microglial metabolic and gene regulation states: the neuroinflammatory and neuroprotective states, respectively. We continue to use these terms in this review article since they are still useful for characterizing microglial states and for evaluating pharmacological efficacy in relation to microglial activity, as described in previous reports discussed below. Nevertheless, we will later discuss this discrepancy *in vivo* between single-cell transcriptomic and histopathological findings.

## Regulatory Role of Ecb on Microglial Polarization in Cell Culture

The eCB system has long been recognized as a modulator of neuronal synaptic activity and the inflammatory response. Our knowledge on the role of eCB in the immune system has rapidly expanded in the last decade, and accumulating evidence shows that the eCB system is deeply involved in regulating polarization phenotypes in microglia. In this section, recent *in vitro* studies regarding microglial modulation by the eCB system are reviewed and summarized (Table 1).

**Table 1:** Effects of eCB modulation on microglial polarization *in vitro*.

**Treatment**	**Cell culture**	**eCB activation method**	**M1/M2 phenotype**	**Other key findings/antagonist tests**	**References**
LPS (1 μg/mL) 24 h	mouse primary MG	BCP (1 μM) 24 h prior to LPS	IL-1β/TNFα/PGE2/iNOS/NO/ROS ↓; IL-10/urea/Arg1/GSH ↑	cell proliferation up; AM630 (1 μM) but not GW9662 (1 μM) reversed	(80)
LPS (100 ng/mL) 8–24 h	BV2	PF3845 (10 μM), URB597 (10 μM) 30 min prior to LPS: FAAH siRNA	PF and URB: PGE2/COX-2/iNOS/IL-6/IL-1β/MCP-1 ↓; IL-10/IL-4/Arg1/Ym1 no change; siRNA: PGE2/COX-2/iNOS/IL-6/IL-1β/MCP-1 ↓; IL-10/IL-4 ↑; Arg1/Ym1 ↑ w/o LPS	SR1/SR2/GW6471/GW9662/O1918 no effect	(81)
LPS (10 ng/mL) + IFNγ (10 U/mL) 3 h	N9	AM1241(10 μM) co-incubated	TNFα/iNOS ↓; Arg1/BDNF ↑	mitochondria/mtDNA/ATP/complex1 &4/Nrf1/Tfam/COX IV/MMP ↑; PGC-1α knockdown reversed	(82)
IFNγ (100 U/mL) + WIN55,212-2 (1 μM) 25 h	BV2	SR1 (1 μM) 1 h post IFNγ/WIN	IL-10 ↓; NO release ↑; MCP-1/TNFα/IL-1β/IL-6/IL-17/IFNγ/CX3CL1 ↑	IL-4/IL-10 ↓ and IFNγ/IL-17 ↑ in CD4+ T cells cultured in BV2 conditioned medium with SR1	(83)
	BV2	VCE004.8 (1 μM) 24 h	Arg1/PPARγ ↑	SR2 no effect; GW9662 no effect	(84)
LPS (25 ng/mL) 24 h	BV2	EEQ-EA (5-10 μM) or EDP-EA (5–10 μM) 4 h prior to LPS	IL-6/nitrite/cytotoxicity ↓; IL-10 ↑	AM630 (1 μM) reversed; eCB metabolites by CYP450 detected in brain; antiangiogenic	(85)
thrombin (20 U/mL) 48 h	rat primary MG	JWH133 (4 μM) 24 h post thrombin	IFNγ/CD86/CD68/IL-1β/TNFα ↓; TGFβ/IL-4/IL-10/CD206/Ym1 ↑	AM630 (1 μM) reversed; PKA inhibitor reversed; cAMP/P-PKA/Epac1 ↑	(86)
LPS (100 ng/mL) 24 h	rat primary MG	AEA (1 μM) 15 min prior to LPS	IL-6/COX-2/iNOS/NO ↓; IL-1β/IL-18/TNFα no change; IL-10/NGF ↑	AM630 but not AM251/CID1602 reversed NO release; AM630 reversed IL-18/TNFα/COX-2	(87)
LPS (50 ng/mL), IL-4 (10 ng/mL) + IL-13 (10 ng/mL), or TGFβ (20 ng/mL) 6 or 24 h	rat, mouse, or human primary MG	2-AG (1 nM) or AEA (1 nM) 24 h	2-AG but not AEA ↑ in M2a (IL-4/IL-13); AEA but not 2-AG ↑ in M2c (TGFβ); Arg1/SOCS3/CB1 ↑ by 2-AG; Arg1/SOCS3/CB2 ↑ by AEA	AM251 (1 μM) and AM630 (1 μM) 0.5 h prior to IL-4/IL-13 reversed Arg1; CB2 KO ↓ Arg1/phagocytosis	(88)
LPS (1 μg/mL) 12 or 24 h	C8B4, human primary MG	SMM-189 1 h post LPS	CD16/32 ↓; CD206 ↑; rod-shape ↑; round/amoeboid shape ↓; eotaxin/IP10/MCP-1/ TARC/MIP-1β ↓	HU308/JWH133: CD16/32 and CD206 ↓; SR2: CD206 but not CD16/32 ↑	(89)
LPS (1 μg/mL) 24 h	BV2, mouse primary MG	JZL184 (1 μM) 1 h prior to LPS; MAGL overexpression	JZL: Fcγ-induced phagocytosis ↓; inflammatory cytokines/iNOS no change (primary MG); MAGL overexpression: Fcγ-induced phagocytosis ↑ (BV2)	phagocytosis Fcγ-mediated; MG132 reversed effects of MAGL; MAGL knockdown no effect	(90)
LPS (10 ng/mL) + IFNγ (10 U/mL) 24 h	N9 MG	AM1241 (5 μM) 1 h prior to LPS	Arg1/IL-10/BDNF/GDNF ↑; iNOS/IL-1β/IL-6/TNFα ↓	AM630 (10 μM) reversed; PKC inhibitor (10 μM) reversed	(91)
LPS (1 μg/mL or 0.1 μg/mL) 18 or 24 h	human primary or immortalized MG	SMM-189 (9.8 μM) 1 h post LPS or (13.4 μM) co-treated with LPS or IL-4	CD11b/CD45/CD80 ↓; IL-8/chemokines/IFNγ/IL-6/IL-12/IL-10 ↓; CD206 ↑ in IL-4 co-treated	LPS/IFNγ/IL-10/IL-4 ↑ CB2	(92)
LPS (50 ng/mL) + IFNγ (100 U/mL) 24 h	mouse primary MG and neuron mix	AEA (10 μM) co-treatment	IL-1β/IL-6 ↓; IL-10 ↑	ERK/JNK signal involved; CD200R ↑; neuron death ↓; CD200R KO and CD200 Ab reversed	(93)
TMEV infection at MOI (5 PFU/cell) 18 or 24 h	mouse primary MG	AEA (10 μM) co-treatment	IL-12/IL-23/IL-17A/NFκB ↓; IL-10 ↑	SR2 (1 μM) not SR1 (1 μM) reversed; Erk/Jnk inhibitor reversed; IL-10 Ab reversed IL-12/IL-23	(94)
LPS (50 ng/mL) + IFNγ (100 U/mL) 24 h	mouse primary MG	AEA (10 μM) co-treatment	NFκB/IL-12/IL-23/P-IκBα ↓; IL-10 ↑	ERK1/2/JNK/NFκB pathways involved; SR2 reversed; AEA treated conditioned medium down T-bet (Th1) but up GATA3 (Th2) in splenocyte	(95)

*↓↑, increased or decreased by eCB treatment, respectively; Ab, antibody; h, hour; KO, knockout mouse; MG, microglia*.

### M2 Polarization Regulated by eCB

Regulation of M2 polarization by eCB and the sub-phenotype characterization of microglia induced by the eCB system have been extensively investigated by Dr. Guaza's laboratory since 2010 (95). When mouse primary microglia activated by LPS and IFNγ were co-incubated with AEA, expression of IL-10 was dose-dependently increased. This gene regulation was likely mediated by CB2, ERK1/2, JNK, and NF-κB but not by PI3K/Akt signaling pathways. In their subsequent report, neurotoxicity triggered by microglia was examined using a mixed culture of neurons and reactive microglia activated by LPS and IFNγ. Treatment with AEA reduced neuron toxicity, downregulated IL-1β and IL-6, and upregulated IL-10 and the CD200 receptor (CD200R), which is known to suppress the microglial inflammatory response and maintain the homeostatic state via interaction with the neuron-derived ligand, CD200 (96). Thus, CD200-CD200R axis enhancement by AEA may underlie its neuroprotective effects, and it may also shift microglial polarization toward the M2 phenotype and/or the homeostatic M0 state (93). In primary microglia activated by infection with Theiler's murine encephalomyelitis virus (TMEV), AEA treatment increased the expression of IL-10 and decreased the expression of the proinflammatory cytokines, IL-12p70 and IL-23. N-arachidonoylserotonin (AA-5HT), an endogenous cannabinoid that inhibits FAAH (97) and TRPV1 (98), also dose-dependently upregulated IL-10 in the TMEV model (94). Moreover, using rat primary microglia without activation, they found that both 2-AG and AEA at 1 nM concentrations were potent inducers of M2 markers, such as Arg1, which increased more than 20-fold (88). Of note, the higher concentration (100 nM) was counteractive to M2 marker induction. When the primary cultures were stimulated with the M2a-subtype inducers, IL-4 and IL-13, 2-AG levels but not AEA levels increased, and the expression of Arg1 and IGF1 increased as well. Administration of TGF-β shifted microglia toward an M2c subtype, indicated by an increase in SOCS3 expression and AEA levels but not 2-AG levels. The eCB metabolic enzymes are distinctly regulated among the two phenotypes: DAGLα was increased while MAGL was decreased in the M2a subtype; NAPE-PLD was increased while FAAH was decreased in the M2c subtype. Upregulation of the biosynthetic enzymes and downregulation of the degrading enzymes resulted in an increase in 2-AG and AEA levels in the M2a and M2c states, respectively. This study clearly demonstrated that the eCB system is tightly regulated by the M2 polarization sub-phenotype, and, in turn, M2 polarization is significantly regulated by endogenous eCB ligands. Consistently, another study showed that M2 polarization by the eCB system is critical under pathological conditions induced by LPS and/or IFNγ. Treatment with AEA in LPS-induced rat primary microglia downregulated IL-6, COX-2, and iNOS and reduced NO production, while IL-10 and NGF were increased dependent on CB2R (87).

Epoxyeicosatetraenoic acid-ethanolamide (EEQ-EA) and epoxydocosapentaenoic acid-ethanolamide (EDP-EA), which are the epoxide derivatives of eCBs catalyzed by CYP450, have recently been discovered in rat brain samples (85). Administration of these metabolites showed decreased IL-6 expression and nitrite production but increased IL-10 expression in LPS-activated BV2 cells; these effects were partially dependent on CB2R activation.

### M2 Polarization Mediated by CB Receptor Agonists/Inverse Agonists

Microglia express both CB1R and CB2R; however, CB2R is more abundantly expressed in microglial cells (99), and its expression is further increased during activation *in vitro* and in disease animal models (23). Therefore, it is expected that CB2R plays a crucial role in the anti-inflammatory microglial response. Upregulation of the alternative M2 markers by CB2R activation in microglial cells has been reported (91). CB2R agonist AM1241 was shown to suppress the expression of proinflammatory cytokines, IL-1β, IL-6, and iNOS, in LPS/INFγ-activated N9 microglial cells. At the same time, there was an increase in the expression of Arg1, IL-10, and the neurotrophic factors BDNF and GDNF, which were significantly reduced by co-administration of the CB2R antagonist AM630 or the PKC inhibitor. In a recent follow-up study, AM1241 also showed an acceleration of mitochondrial biogenesis and an increase in the expression of mitochondrial transcription factors and genes, such as Nrf1, Tfam, and cytochrome *c* oxidase subunit IV, under the control of PPARγ coactivator-1α (PGC-1α) (82). This finding is quite intriguing given that the metabolic balance of energy in microglia/macrophages is drastically switched from the glycolytic pathway in the M1 state to the mitochondrial respiratory pathway in the M2 state (100). Mitochondrial biogenesis could be one aspect of M2 polarization regulation by eCB that could enhance mitochondrial oxidative phosphorylation. M2 phenotype modulation by eCB was observed in cells under other pathological conditions, including cells from an intracerebral hemorrhage model (86). Treatment with JWH133 upregulated the expression of several M2 markers, such as TGF-β, IL-4, IL-10, CD206, and Ym1, in microglial cells. M2 marker upregulation was reversed by the CB2R antagonist AM630. Other research groups tested the effect of a natural CB2 agonist, β-caryophyllene (BCP), which has been approved by the FDA as a food additive. IL-1β and TNFα were downregulated, and iNOS expression and ROS production were reduced in mouse primary microglia when 1 μM but not 5 μM of BCP was added 24 h prior to LPS treatment. In contrast, IL-10 and Arg1 were upregulated. As a result of increased Arg1 and decreased iNOS, generation of urea was increased, while that of NO was reduced. These changes clearly indicate that the metabolic balance of arginine was shifted toward the M2 phenotype (80). VE004.8 is a dual agonist for PPARγ and CB2R. Navarrete and colleagues investigated the pharmacological effects of this compound using several different cell types, including endothelial cells, RAW264.7 macrophages, and BV2 microglial cells, in a hypoxic environment (84). Results from the experiments using RAW264.7 and BV2 cells showed a consistent increase in Arg1 and PPARγ, and this increase was not affected by co-administration of the PPARγ antagonist, GW9662. Although CB1R expression is very low in microglia, its activation has also been reported to modulate microglial polarization (83). When BV2 cells activated with IFNγ were co-incubated with SR141716A, a CB1R antagonist, the expression of TNFα, IL-1β, and IL-6 were upregulated. In addition, when IFNγ-activated BV2 cells were incubated with the CB1R/CB2R agonist WIN55212-2, co-incubation with SR141716A reduced the expression of IL-10 and increased the expression of inflammatory cytokines and Cx3cl1. Thus, CB1R-mediated modulation may also trigger a shift toward the alternative phenotype in microglia under certain culture conditions.

CB2R signaling blockade by inverse agonists does not always induce the inflammatory outcome; in fact, it has been shown to induce the anti-inflammatory response in some situations. A synthetic CB2R-selective inverse agonist, SMM-189 (101), was reported to downregulate the expression of several M1 markers, including CD16/32, IFNγ, IL-6, IL-8, and MCP-1, while upregulating M2 markers, such as CD206 and CD209 (89, 92). Of note, the microglia (C8B4 cells) that they used behaved in a contradictory manner compared to other reports; CD206 expression was upregulated by SR144528, a CB2R antagonist, but downregulated by the CB2R agonists JWH133 and HU308. In their earlier report, CD206 was increased by SMM-189 in the presence of IL-4 in human microglial cells, while IL-10 was decreased in primary human microglia (92). Thus, microglial M2 polarization by SMM-189 seems unclear in *in vitro* culture systems.

### M2 Polarization Modulated by eCB Degradation Inhibitors

Inhibition of eCB-degrading enzymes can boost eCB signaling by increasing the levels of endogenous ligands that are available to suppress inflammation, but it is unclear how microglial polarization is altered. We recently reported on the anti-inflammatory effects of PF3845 and URB597, two commonly used FAAH inhibitors, and FAAH knockdown by siRNA in BV2 cells. Both pharmacological and genetic inhibition downregulated COX-2, iNOS, and proinflammatory cytokine expression; however, only siRNA knockdown of FAAH showed enhancement of M2 markers, such as IL-4 and IL-10, both in the presence and absence of LPS treatment (81). The discrepancy between the pharmacological inhibition and siRNA knockdown is unclear; however, different downstream pathways might be involved. Until now, there have been only a few studies examining the effects of eCB-degrading enzyme inhibitors on microglial alternative activation *in vitro*, and future studies in this area may help illuminate the reason for these inconsistencies.

## Effects of eCB Modulation on Microglial Polarization in Animal Models

Microglia are one of the central players in neuroinflammation linked to many neurological diseases (60, 102, 103). A substantial number of studies have examined whether modulation of microglial/macrophage activation was affected by the eCB system in preclinical animal studies. Herein, we review microglial polarization by eCB in animal models of neurodegenerative diseases, such as Alzheimer's disease (AD), Parkinson's disease (PD), multiple sclerosis (MS), traumatic brain injury (TBI), and stroke related diseases. The results of these studies are briefly summarized in Table 2. Readers are encouraged to refer to several excellent review articles recently published regarding microglial activation and its potentially pathogenic role in AD (120), PD (121), and TBI (5, 122).

**Table 2:** Effects of eCB modulation on microglial polarization in animal disease models.

**Model construction/ animal**	**Disease**	**eCB treatment**	**M1/M2 phenotype**	**Other pathologies**	**Behavioral test**	**Antagonist test**	**References**
Female SJL/J (4–6 wk), TMEV (2 ×10^6^ PFU) right intracerebral injection	MS	2-AG (3.5 mg/kg) subcutaneous 28DPI daily	35DPI: Iba1 IR/TNFα/Il-1β/IL-10/ Cxcl12 ↑; CD206/CD68 no change; MBP-loaded MG no change 42DPI: Cxcl12/BDNF ↑; MBP-loaded MG ↑; CD206/CD68 no change	35DPI: Sema3F/Napepld/Msr1 ↑, MBP/PLP/Olig2/CC1/OPC/Ccl2/CD47/ Mgf-e8 no change; 45DPI: Sema3A/Sema3F/Napepld/ Lamp1 ↑; CD47/Sirp1a/Mgf-e8 no change; Faah ↓; 60DPI: g-ratio ↓; CC1^+^/OPC/MBP/ PLP ↑; Iba1^+^ ↓			(104)
Male C57BL/6 (3 mo), air blast (50 psi) left side	TBI	raloxifene (5 or 10 mg/kg) i.p. 2 h post TBI + daily for 14 days	Iba1^+^ ↓; CD16/32^+^ ↓; CD206^+^ ↑ in right optic nerve; M1/M2 ratio ↓	Optic nerve axon count ↑; occulomotor nucleus loss ↓	visual activities improved		(105)
Male C57BL/6 (3 mo), air blast (50 psi) left side	TBI	SMM-189 (6 mg/kg) i.p. 2 h post TBI + daily for 14 days	CD16/32^+^ ↓; CD206^+^ ↑ in optic tract; Iba1^+^ ↓ in retina	Axon bulbs ↓ in optic tract; optic nerve ↑; GFAP^+^ ↓ in retina	visual function (contrast sensitivity) improved		(106)
Male APP/PS1 TG (8 mo)	AD	JWH015 (0.5 mg/kg) i.p. for 8wk daily	Iba1 IR/IL-6/TNFα/iNOS ↓; Ym1/2 ↑ in crtx but no change in hipp	Plaque # no change; dendritic spine complexity ↑ in cortex but no change in hipp	no change Morris water maze; improve novel object recognition		(107)
Female SJL/J (4-6wk), TMEV (2 ×10^6^ PFU) right intracerebral injection	MS	2-AG (5mg/kg) or UCM-03025 (5 mg/kg) i.p. for 7 days	Arg1^+^ CD11b^+^ (MG/macrophage) ↑; activated MG morphology ↓; Iba1^+^ ↓; Arg1/IL-10/IFNγ ↑; iNOS/TNFα/IL-1β/ Ccr2/Ccl2/Ccl3/ICAM1/Csf1r ↓	CD45^+^ infiltration ↓; Arg1^+^ Iba1-(M-MDSC-like) ↑; CD4^+^ T cell ↓; viperin/Bax/Casp3 ↓; Bcl2 no change	vertical motor activity improved	AM630 reversed more than AM251	(108)
Male S-D rat (300–400 g), vertebral artery occlusion and 10 min ischemia 24 h later	Four-vessel occlusion induced vascular dementia	Paeoniflorin (40 mg/kg) or HU308 (3 mg/kg) i.p. 15 min post occlusion then daily for 27 day	IL-1β/TNFα/IL-6/CD68^+^/nitrite/iNOS/ NF-κB/IκBα/mTOR ↓; IL-10/TGF-β1/Arg1/YM1/CB2/ CD206^+^ ↑	Neuronal damage in hipp CA1 ↓; P-IκBα/P-mTOR↓; P-PI3K/P-Akt ↑	spatial memory improved	AM630 reversed	(109)
Male strain unknown (8–10 wk), PMCAO (MCA cauterized) 24 or 48 h	stroke	JZL184 (4 mg/kg) i.p. immediately after PMCAO	TNFα/MMP9 ↓; IL-10 ↑	Infarct/edema ↓; improved neurological damage (bederson test)	sensorimotor function/muscle performance/ neurological deficit score improved	AM251 (3 mg/kg) did not reverse but improved some tests	(110)
Male C57BL/6 or CD-1 (12–16 wk), CCI 3 mm depression X 3 mm diameter convex tip	TBI	GP1a (3 mg/kg) or AM630 (5 mg/kg) i.p. 10 min post CCI	iNOS/TNFα/IL-6/IL-1β/Ccl2/Cxcl10 ↓; IL-10/Arg1 ↑; M1 type (CD68^+^ TNFα^+^ CD206-) ↓; M2 type (CD68^+^ IL10^+^ CD206^+^) ↑ in macrophage/microglia (CB2^+^/CD11b^+^)	Edema ↓; cerebral perfusion ↑; CD45high macrophage ↓; CD45low MG no change	motor function/anxiety improved	AM630 no effects	(111)
Male S-D rat (250–300 g), 100 μl autologous arterial blood infusion into right basal ganglia	Intracerebellar hemorrhage (stroke)	JWH133 (1.5 mg/kg) i.p. 1 h post-surgery	Arg1/Ym1/CCL22/CD206/IL-4/IL-10/ TGFβ ↑; CD32/CD86/CD68/IL-1β/ iNOS/TNFα ↓	Apoptotic/damaged neuron ↓; P-CREB/P-PKA ↑; edema ↓	Neurological severity score/forelimb placing test improved	SR2 (3 mg/kg) i.p. 3 min prior JWH reversed; CREB KD reversed	(112)
Male S-D rat pup P7, 0.3 U bacterial collagenase infusion to right ganglionic eminence	GMH (stroke)	JWH133 treatment for 7 days	Iba1^+^/BDNF/ramified MG/CX3CR1 ↑	BrdU^+^ neuron/MAP2/nestin/NeuN/ Tuj-1/NeuroD ↑; fiber bundle up in internal capsule		NeuN/NeuroD/Tuj-1/Dbcn/Iba1^+^/NeuroD^+^ ↓ by CX3CR1 knockdown	(113)
Male S-D rat pup (15–7 g), 0.3 U bacterial collagenase infusion to right ganglionic eminence	GMH (stroke)	JWH133 (1 mg/kg) 1 h post infusion i.p. 3–72h	CD68^+^/CD68/CD86/iNOS/IFNγ/IL-1β/ TNFα ↓; CD206^+^/CD206/Arg1/Ym1/IL-4/IL-10/ BDNF ↑			AM630 (1 mg/kg) reversed	(86)
Female C57BL/6 (8–10 wk), MOG35-55 (300 μg) subcutaneous injection	MS	CB1 Lentivirus (1.75 ×10^8^ TFU) intrathecal injection in lumbar spinal cord 5 day prior to EAE	NFκB/Tlr4/IL-1β/IL-6/TNFα/IL-17A ↓; IL-10/NT3/BDNF/GDNF ↑; IFNγ no change in spinal cord at 28dpi	Demyelination/infiltration ↓; IFNγ^+^/IL-10^+^/IL-17^+^ no change in CD4^+^Tcells; CD206^+^/IL-10^+^ ↑ but CD16/32^+^ ↓ in splenic CD11b^+^ monocyte	Clinical score ↓		(114)
Male C57BL/6 (3 mo), MPTP hydrochloride (20 mg/kg) and probenecid (250 mg/kg) twice/week for 5 wks i.p.	PD	JZL184 (8 mg/kg) i.p. 5 d/wk for 5 wks	TGFβ/GDNF ↑ but no change IL-1β/ IL-6/TNFα in Strtm; Iba1^+^; ramified/small cell body sized MG ↑ in Strtm	2-AG/AEA ↑ in midbrain; TH^+^ ↑ not MAC1^+^ in SNpc; DAT/TH/TH^+^/GFAP^+^/Iba1^+^ ↑ in strtm; cytoplasmic b-catenin ↓; nuclear b-catenin ↑ in Strtm	Improved motor function in MPTP model		(115)
Male C57BL/6 (8 wk), CCI 3 mm diameter X 1.5 mm depth left parietal crtx once	TBI	PF3845 (5 mg/kg) i.p. 30 min post CCI + daily	COX-2/COX-2^+^/iNOS/iNOS^+^ ↓ in crtx and hipp; Arg1^+^ ↑ in crtx	AEA/2-AG/Synaptophysin/Bcl2/ Hsp70/Hsp72 ↑; lesion volume/ damaged neuron/APP ↓ in crtx; P-ERK/P-AKT ↑	Improved memory/fine motor skills/ anxiety	AM281 (3 mg/kg) and AM630 (3 mg/kg) reversed neuron damage/motor function/working memory	(116)
Male C57BL/6 (8 wk), CCI 3 mm diameter X 1.5 mm depth left parietal crtx once	TBI	WWL70 (10 mg/kg) i.p. 30 min post CCI + daily	iNOS/iNOS^+^/COX-2/COX-2^+^ ↓; Arg1^+^ ↑	Lesion volume ↓; degenerated neurons ↓; BBB breakdown ↓; P-AKT/P-ERK/CB1/CB2 ↑	Motor function and working memory improved	AM281 (3 mg/kg) and AM630 (3 mg/kg) reversed neuron damage	(117)
Male Swiss (8–10 wk), permanent MCAO ligature of trunk before bifurcation for 15–24 h,	stroke	JWH133 (1.5 mg/kg) i.p. 10 min post MCAO	Iba1^+^/IL-6/IL-12/MIP-1α/MCP-1/ RANTES/iNOS ↓; IL-10/TGFβ/Ym1 ↓; COX-2/MPO/Arg1/IL-4 no change	Infarct ↓	Neurological severity score ↓ after 48 h	SR2 (3–5mg/kg) i.p. 3 min prior JWH reversed, CB2KO no effect of JWH133	(118)
Female SJL/J (4–6 wk), TMEV (10^6^ PFU) right intracerebral injection	MS	AEA (3.5 mg/kg) infusion 83DPI for 7 days; AA-5HT (5 mg/kg) 78DPI for 12 d	IL-1β/IL-6/IL-12/IL-23/IL-17A ↓; IL-10 ↑ in serum/spinal cord	CD200/CD200R1 ↑	Improved motor function		(93)
Female SJL/6 (6–8 wk), PLP (150 μg) subcutaneous injection	MS	2-AG (100 μg) i.p. from D0 for 14 days	Ramified MG ↑; Arg1^+^ ↑; iNOS^+^ no change 22DPI	Axonal loss ↓; lymph node cells ↓; CB1/CB2 ↑			(119)

*↓↑, increased or decreased by eCB treatment, respectively; crtx, cortex; DPI, days post injury; h, hour; hipp, hippocampus; i.p., intraperitoneal injection; IR, immunoreactivity; KD, knockdown; KO, knockout mouse; MG, microglia; MOI, multiplicity of infection; mo, month; P7, postnatal day7; PFU, plaque-forming unit; psi, pounds per square inch; S-D rat, Sparague-Dawley rat; SNpc, substantia nigra pars compacta; strtm, striatum; TG, transgenic mouse; wk, week*.

### Microglial Polarization by eCB in MS-Related Animal Models

To our knowledge, the first reports of microglial M2 polarization by the eCB system in *in vivo* studies were published by Dr. Guaza's group (94) and Dr. Simeonidou's group (119). Using an experimental autoimmune encephalopathy (EAE) model created by PLP injection, Simeonidou's group studied the modulatory effects of 2-AG in the EAE model and showed that administration of 2-AG increased the number of ramified microglia, which resemble homeostatic microglia, and the number of Arg1^+^ cells; however, the iNOS^+^ cell population was unchanged (119). Consistent with *in vitro* culture studies (see above), administration of AEA reduced the expression of several proinflammatory cytokines, increased that of IL-10 in serum at 90 days post infection (dpi), and improved motor function in a demyelinating disease model induced by TMEV (94). In a recent study, the effects of 2-AG on microglial polarization were examined in the early phase of TMEV induction (7 dpi) (108). Proinflammatory cytokines, including IL-1β, TNFα, IFNγ, and iNOS, as well as chemokines and chemokine receptors, including Ccr2, Ccl2, Ccl3, and Ccl5, were substantially reduced by 2-AG administration. In contrast, the expression of Arg1 and IL-10 was increased several-fold. It was found that in addition to an increase in Arg1^+^/CD11b^+^ microglia/macrophages, the number of Arg1^+^/Iba1^−^ cells, which are putatively monocytic-myeloid derived suppressor cells (M-MDSCs) that have infiltrated the CNS, also increased. These results suggest that increased Arg1 expression was derived not only from microglia but also from infiltrated macrophages or M-MDSCs (108). In a recent study (104), 2-AG was subcutaneously injected into a TMEV animal model during the late stage of the infection (28 dpi) for 1 or 2 weeks. With this regimen, the pathological signature of demyelination [i.e., loss of CC1^+^ cells, reduced myelin basic protein (MBP), and a high g-ratio] was significantly ameliorated after 60 days; however, modulation of the M2 phenotype was unclear: TNFα, IL-1β, and IL-10 were all upregulated after 35 days. Expression of both CD68 and CD206 were unchanged after both 35 and 42 days. The only gene associated with the M2 phenotype that was significantly altered after 42 days was BDNF, which was increased. This study implies that M2 phenotype polarization and pathological profile are not always correlated with each other (104). Although it is generally believed that CB2R plays a key role in the anti-inflammatory effects of the eCB system, CB1R overexpression in lumbar spinal cord delayed the onset of clinical symptoms and attenuated clinical score and demyelination in an MS model induced by MOG peptide immunization (114). Proinflammatory genes, including TLR4, IL-1β, IL-6, IL-17, and TNFα, and the key transcription factor NF-κB were downregulated specifically in the spinal cord but not in the brain and spleen. In contrast, there was an increase in IL-10^+^ and CD206^+^ microglial/macrophage cells and an increase in the neurotrophic factors NT3, BDNF, and GDNF in the spinal cord. These phenotypic changes indicate the potential for neuroprotective effects and axon repair. Recently, their follow-up study indicated that the use of SR141716A exacerbated EAE clinical scores and upregulated the expression of NF-κB and proinflammatory cytokines and chemokines. This finding was consistent with the notion that CB1R activation plays a key role in the eCB anti-inflammatory response in the MS model (83).

### Microglial Polarization by eCB in Cerebral Hemorrhage and Stroke Models

Germinal matrix hemorrhage (GMH) is defined as damage to the brain resulting from the rupture of blood vessels within the subependymal germinal region of the ganglionic eminence in the immature brain. Neuroinflammation is deeply involved in the disease pathogenesis and progression. The therapeutic effects of the CB2 agonist JWH133 on microglial activation in the GMH animal model, which was created by intracerebral infusion of collagenase, have been extensively investigated. After a 24 h infusion, JWH133-administered animals showed attenuated edema and perihematomal tissue injury and improved motor and memory function (123). In addition, Iba1^+^ and reactive microglia populations were reduced (124). In a subsequent report, they characterized time-dependent changes in M1 markers in perihemotomas and found that upregulation of M1 marker (IFNγ, IL-1β, TNFα, CD68, CD86, and iNOS) expression started relatively early (6–24 h post-injury) and was attenuated by JWH133. In contrast, regulation of the expression of M2 markers (IL-4, IL-10, BDNF, Arg1, Ym1, and CD206) was slightly delayed (24–72 h post-injury) and was enhanced by JWH133 (86). Thus, as shown in other brain injury models, microglial activation of the M1 phenotype was induced early on (6 h post-injury) and then downregulated, while that of the M2 phenotype was induced at a later time point with a potential peak at 24–72 h post-injury. This study demonstrated that the eCB system modulates both M1 and M2 marker expression in a time-dependent manner in the brain injury model. In a later study, they examined the effects of long-term treatment with JWH133 in the disease model and found that microglia adopted a ramified cell shape and showed increased expression of CX3CR1, the fractalkine receptor (113). CX3CR1 is not regarded as an M2 marker; however, upon binding to the neuron derived ligand, CX3CR1 suppresses microglial activation and enables microglia to return to the homeostatic state. These studies suggest that the CB2 agonist may shift the microglial phenotype to either the alternative (M2) or the homeostatic (M0) state depending on the experimental settings. The therapeutic effect of JWH133 was also investigated in another hemorrhage model created by the infusion of arterial blood into the basal ganglia (112). In this model, JWH133 was found to reduce brain edema, neurological scores, neurodegeneration, and apoptotic neuronal cells. M1 markers, including TNFα, IL-1β, CD68, and CD32, were all suppressed throughout the time period from 6 to 72 h post-injury, while M2 markers, including Ym1, Arg1, IL-4, IL-10, and TGF-β, were enhanced 24 h post-injury. Interestingly, since downregulation of phosphorylated CREB in the disease animal was reversed by JWH133, they created a knockdown animal model of CREB via intracerebroventricular infusion of siRNA and found that CD68 expression was upregulated and CD206 expression was downregulated in perihematomal tissue from the JWH133-treated animals. These results suggest that microglial classical (M1) and alternative activation (M2) marker expression may be regulated by CREB-mediated signal transduction (112).

Another study used a permanent cerebral ischemia model to examine the MAGL inhibitor, JZL184. Disease pathology, such as edema and infarct volume, was reduced by treatment with JZL184. In addition, TNFα and MMP9 expression was downregulated, while IL-10 expression was upregulated in the JZL184-treated group compared to the vehicle control. Interestingly, co-administration with CB1R antagonist AM251 did not significantly reverse the effects of JZL184 in some behavioral tests and pathologies (110). Paeoniflorin, an active ingredient in a traditional Chinese medicine, was reported to be a CB2R agonist that modulates the M2 phenotype evidenced by an increase in M2 markers, including Arg1, Ym1, IL-10, and TGF-β1, and a decrease in M1 markers, including IL-1β, IL-6, and TNFα, in a middle cerebral artery occlusion (MCAO) model. These modulatory effects were reversed by co-treatment with AM630 (109). However, it is unknown whether this compound is a CB2R agonist, despite its ability to increase CB2R expression. Another study examined the effects of JWH133 in the MCAO model (118). Intraperitoneal injection of JWH133 10 min prior to occlusion improved infarct volume and neurological severity score after 48 h. Several M1 markers tested (i.e., IL-1β, IL-6, iNOS, TNFα) and Iba1^+^ cells in the ipsilateral region were suppressed between 15 and 24 h. In addition, M2 markers, including IL-10, Ym1, and TGF-β, were also downregulated, whereas Arg1 and IL-4 expression was not significantly changed. The authors hypothesized that CB2R activation shifts microglia toward the inactivated state and results in anti-inflammation (118).

### Microglial Polarization by eCB in Neurodegenerative Disease Models

A plethora of studies have shown that microglial activation can be the cause of neurotoxicity and the development of neurodegenerative diseases such as AD, PD, and ALS. Therefore, several preclinical studies have examined potential treatments, including eCBs, to suppress microglial neuroinflammation in these disease models (125). However, only a few investigations have examined the role of eCB system modulation on microglial polarization. In a recent study using the APP/PS1 transgenic AD mouse model, administration of the CB2 agonist JWH015 for 8 weeks significantly decreased the expression of Iba1^+^ cells and proinflammatory cytokines and increased the expression of YM1/2 in the cortex (107). However, anti-inflammatory effects were not observed in the hippocampus. In line with these microglial responses, performance in the novel object recognition test associated with the cortex was improved, whereas performance in the Morris water maze test related to hippocampal spatial memory was not significantly improved. These results suggest that the CB2 agonist modulates microglial phenotype in a region-dependent manner. Aymerich's group studied the effects of JZL184, a MAGL inhibitor, on the MPTP-induced PD model (115). After intraperitoneal injection of JZL184 for 5 days a week over 5 weeks, dramatically decreased dopamine active transporter (DAT) and tyrosine hydroxylase (TH) expression in the PD animal was partially but significantly reversed. Moreover, TH+ neurons in the SNpc were increased. The number of Iba1^+^ microglia with longer ramifications and a larger cell body increased. Neuroprotective striatal TGF-β and GDNF expression was increased, but inflammatory cytokines, such as IL-1β, IL-6, and TNFα, were not significantly changed. The observed upregulation of TGF-β and GDNF is not necessarily derived from microglia alone but also possibly from astrocytes since GFAP immunoreactivity in the region was significantly increased. Since β-catenin levels in the nucleus were increased, Wnt/catenin signaling may be also involved in the anti-inflammatory response by JZL184. Two behavioral tests, the pole test and the rotarod test, showed that motor function was improved by JZL184 treatment in the MPTP model but not in control animals (115).

### Microglial Polarization by eCB in TBI Models

Two research papers regarding microglial polarization by eCB in a TBI model have been published by our laboratory. Our TBI model was created by controlled cortical impact (CCI), and two different inhibitors of eCB-degrading enzymes were tested. In the first paper, injection of WWL70, an inhibitor for ABHD6, which is one of the enzymes responsible for hydrolyzing 2-AG, was administered 30 min after the initial injury and then once a day until the end of session (117). The TBI animals showed memory deficits, motor dysfunction, a pathologically massive tissue lesion, and blood brain barrier breakdown. WWL70 not only attenuated these behavioral impairments and brain pathologies, but it also suppressed the expression of COX-2 and iNOS and dramatically increased Arg1 expression. These results indicate that the microglial phenotype was shifted to the M2 phenotype by WWL70. In the second paper, we examined PF3845, an inhibitor of FAAH, which is the main hydrolyzing enzyme of AEA in the CNS (116). TBI model animals were injected with the inhibitor in the same manner as described above. One and two weeks after surgery, working memory and motor coordination were improved by PF3845 treatment, and lesion volume and neurodegenerative neurons were reduced. These effects were likely mediated by both CB1R and CB2R. Moreover, COX-2^+^ cells and iNOS^+^ cells were reduced, and Arg1^+^ cells were increased in the ipsilateral cortex by PF3845 treatment. The increase in Arg1 was found at 3 days and continued for at least 2 weeks post-injury. Thus, the two eCB-degrading enzyme inhibitors demonstrated therapeutic efficacy and the potential to modulate microglial phenotype. Our recent report shows that WWL70 inhibits not only ABHD6 but also prostaglandin E synthesis in BV2 microglia (126); these results suggest that eCB-independent mechanisms might also contribute to the therapeutic effect of WWL70 in the TBI mouse model. Another study examined the effects of SMM-189, a CB2R inverse agonist, on microglial phenotype in a TBI mouse model. Consistent with the *in vitro* study, CD16/32^+^ cells were decreased while CD206^+^ cells were increased by SMM-189 administration in the right optic tract 3 days after blast injury (106). Very recently, the same group showed that raloxifene, which is a CB2R inverse agonist (127) but also known as a selective estrogen receptor modulator (128), induced anti-inflammatory effects by modulating the M1/M2 microglial phenotype (105). However, whether microglial modulation is dependent on eCB has not been examined. In a recent study, CB2R agonist GP1a was examined in a TBI model induced by CCI (111). TBI-induced edema, anxiety, and motor dysfunction were ameliorated at 3 mg/kg of GP1a and to a lesser degree at 5 mg/kg. Moreover, CB2R activation by GP1a decreased Ccl2, Cxcl10, iNOS, TNFα, IL-6, and IL-1β and increased IL-10 and Arg1. The CD45^low^ microglia population was unchanged by either TBI or GP1a treatment, whereas CD45^high^ macrophage infiltration induced by TBI was reduced at 3 days post injury. When fluorescence-labeled macrophages were administered intravenously, CB2R immunoreactivity after TBI was correlated with increased fluorescence; this correlation suggests that the cells expressing CB2R in the CNS are mainly macrophages. Based on these observations, it was postulated that mainly the infiltrated macrophages are responsible for the increase in M2 marker expression by CB2 activation; however, the contribution of microglia cannot be dismissed (111).

## Perspectives and Future Issues

A significant number of studies that examine microglial polarization by the eCB system have emerged in the last decade (Table 1 for *in vitro* studies and Table 2 for animal studies). Several studies showed that the therapeutic effects of the eCB system were mediated by CB1R, CB2R, non-canonical receptors GPR55/GPR18, and PPARs; however, CB2R activation is thought to play an indispensable role in eCB-mediated anti-inflammatory effects in several diseases models. Moreover, in terms of microglial phenotypic modulation, CB2R is the predominant regulator both *in vitro* and in animal models.

**Figure 3:**
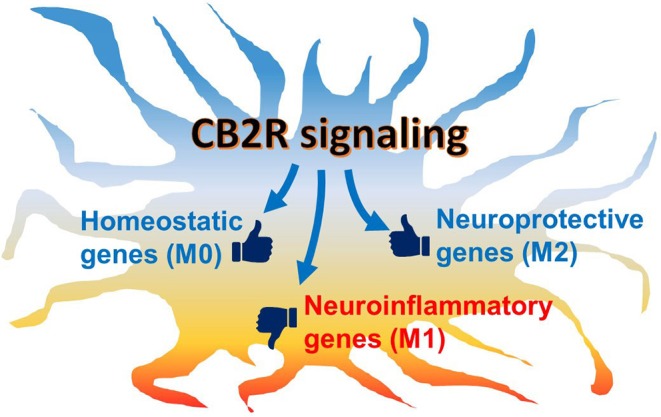
Schematic hypothetical CB2R signaling effects on microglial phenotype. Microglia are activated under neuropathological conditions, in which neuroinflammatory genes are mainly induced while neuroprotective genes are also regulated. Under chronic conditions, the predominantly inflammatory activated microglia often cause neurotoxicity and neurodegeneration. When CB2R, which is abundantly expressed in microglia, is activated, its downstream signaling modulates the balance of gene regulation toward the neuroprotective function. In addition, homeostatic genes, which are associated with communication with neurons and synaptic activity, are also up-regulated to return the cell to physiological conditions.

However, it is still unclear whether CB2R signaling, indeed, has the potential to commit microglia to the alternative (M2) phenotype *in vivo* because of technical limitations in the current approaches and the limited data available: commonly used methods, including gene or protein expression analyses or immunohistochemistry, provide a “snapshot” of the microglial activation state, but tracking changes at the individual cell level remains difficult. Therefore, whether classical activation (M1)-committed microglia can switch to the alternative activation (M2) phenotype or vice versa during disease development or drug intervention is still unknown. It may be possible to address this issue by monitoring live cells in animals using two photon microscopy and genetically labeled or manipulated animals with fluorescent markers, such as Tmem119-EGFP transgenic mice (129–131). Second, the expression analysis of M2 gene markers is useful for alternative (M2) phenotype assessment. However, most of the studies described here have shown only a few markers positively upregulated. Therefore, it is uncertain if the upregulation of only a limited number M2 markers really indicates an acquired commitment to the alternative (M2) phenotype or if it indicates only a partial transition. More comprehensive investigation is needed in order to understand alternative (M2) phenotype modulation. In the last couple of years, several studies have used single-cell RNA-sequencing to investigate individual microglial gene regulation. These studies have consistently demonstrated the heterogeneity of microglial populations dependent on region, age, and pathological conditions (132). Although reactive microglial gene signatures that were evoked by immunostimulation and disease have been identified, none of them have matched the gene set of M2 markers (74–77). To fill the gaps in our understanding of microglial gene expression and subsets, we suggest further investigation, including pathohistological analysis with a stricter classification protocol using microglia-specific markers (i.e., CD45^low^, Tmem119, and P2ry12) and multiple M2 markers rather than only one or two. Nevertheless, although the pure microglial phenotypes can be observed *in vitr*o, the M1/M2 dichotomy is not pathophysiologically relevant since microglia in the brain would never receive only one cytokine but, rather, several environmental cues that modulate their phenotype in either direction. In fact, it was reported in some studies that both M1 and M2 gene markers are co-expressed in the same cells (73, 78). We hypothesize that individual microglia co-express neuroinflammatory (M1-type) genes and neuroprotective (M2-type) genes. Thus, eCB would not switch the microglial population from the M1-like phenotype to the M2-like phenotype; rather, eCB, together with microenvironmental cues, would shift the balance of expression between the two gene sets toward the neuroprotective function (Figure 3). In the future, further investigations, including transcriptomic studies, may reveal new gene markers for the neuroinflammatory M1 and the neuroprotective M2 gene sets.

In terms of morphological changes during microglial polarization, several studies using different animal models (113, 115, 119, 123) have shown that microglial morphology changes to a more ramified cell shape rather than a bipolar or amoeboid shape in disease models after eCB treatment. The latter morphology is thought to be related to classical (M1) activation though microglia have a ramified cell shape with a small soma when in the homeostatic (M0) state. In line with the morphological data, studies showed that eCB administration increased the expression of CD200R *in vitro* in a mixed neuron/microglia culture (93) and CX3CR1 in a stroke model (113), both of which are thought to be associated with alternative (M2) and homeostatic (M0) states (133). Although it remains uncertain whether the increase in homeostatic microglia is merely an epiphenomenon of terminated neuroinflammation, eCB signaling may directly shift microglial morphology toward not only the neuroprotective (M2) phenotype but also the homeostatic (M0) phenotype, in which microglia are known to have important physiological functions, which include synaptic pruning, synaptic plasticity modulation, and neuronal trophic support (46). The homeostatic state induced by eCB may play a role in neuron repair and restore synaptic activity, similar to the putative function of the neuroprotective (M2) phenotype. Further studies are necessary to elucidate the molecular mechanisms of microglial modulation by eCB and to define the classification of microglial phenotypes, including the homeostatic (M0) state, under pathophysiological conditions.

## Author Contributions

MT and SS contributed to literature search, writing and editing the manuscript, and preparing tables and figures. YZ contributed to writing and editing the manuscript.

### Conflict of Interest

The authors declare that the research was conducted in the absence of any commercial or financial relationships that could be construed as a potential conflict of interest.

## Abbreviations

AA-5HT: N-arachidonoylserotonin
Aβ: amyloid beta
Abhd 6/12: α-β-hydrolase domain 6/12
AD: Alzheimer's disease
AEA: anandamide
2-AG: 2-arachidonoyl glycerol
ALS: amyotrophic lateral sclerosis
APP/PS1: amyloid beta precursor protein/presenillin1
Arg1: arginase 1
Bax: Bcl2 associated X
BBB: blood brain barrier
Bcl2: B-cell lymphoma 2
BCP: beta-caryophyllene
Bdnf: brain-derived neurotrophic factor
BrdU: bromodeoxyuridine
Casp3: Caspase 3
CB1/2R: cannabinoid 1/2 receptor
CCI: controlled cortical impact
Ccr: C-C motif chemokine receptor
Ccl: C-C motif chemokine ligand
CD: cluster of differentiation
CNS: central nervous system
COX IV: cytochrome *c* oxidase subunit 4
Cox2: cyclooxygenase2
Creb: cAMP response element binding protein
Cx3cr1: CX3C receptor 1
Cxcl: C-X-C motif chemokine ligand
CYP450: cytochrome P450
Daglα/β: diacylglycerol lipase α/β
DAMPs: Danger Associated Molecular Patterns
DAT: dopamine active transporter
Dbcn: doublecortin
dpi: days post infection
EAE: experimental autoimmune encephalopathy
eCB: endocannabinoid
EEQ-EA: epoxyeicosatetraenoic ethanolamide
EDP-EA: epoxydocosapentaenoic ethanolamide
ERK: extracellular signal-regulated protein kinase
Faah: fatty acid amide hydrolase
Fizz1: found in inflammatory zone 1
Gdnf: glial cell-derived neurotrophic factor
Gfap: glial fibrillary acidic protein
GMH: germinal matrix hemorrhage
GPR: G protein-coupled receptor
HSP: heat shock protein
Iba1: ionized calcium binding adaptor molecule 1
Icam1: intercellular adhesion molecule 1
Igf1: insulin-like growth factor 1
Ifnγ: interferon γ
IL: interleukin
IκBα: nuclear factor of κ light polypeptide gene enhancer in B-cells inhibitor α
iNOS: inducible nitric oxide synthase
JNK: c-Jun NH2 terminal kinase
lamp1: lysosomal-associated membrane protein 1
LPS: lipopolysaccharide
Mac1: macrophage-1 antigen
Magl: monoacylglycerol lipase
MAPK: mitogen-activated protein kinase
Mbp: myelin basic protein
MCAO: middle cerebral artery occlusion
Mcp-1: Ccl2
M-MDSC: monocytic-myeloid derived suppressor cells
MHCII: major histocompatibility complex II
Mip-1α/β: macrophage inflammatory protein-1 α/β
Mmp9: matrix metallopeptidase 9
Mog: myelin oligodendrocyte glycoprotein
Mpo: myeloperoxidase
MS: multiple sclerosis
Msr1: macrophage scavenger receptor 1
mtDNA: mitochondrial deoxyribonucleic acid
Nape-pld: N-acyl phosphatidyl ethanolamine phospholipase D
NF-κB: nuclear factor κ light-chain-enhancer of activated B cells
Ngf: nerve growth factor
NK cells: natural killer cells
NLR: NOD-like receptor
Nrf1: nuclear respiratory factor 1
NT3: neurotrophin 3
OPC: oligodendrocyte precursor cell
PAMPs: Pathogen Associated Molecular Patterns
PD: Parkinson's disease
Pgc-1α: Pparγ coactivator 1-α
PGE2: prostaglandin E2
Plp: proteolipid protein
PMCAO: permanent middle cerebral artery occlusion
PNS: peripheral nervous system
Ppar: peroxisome proliferator-activated receptor
PRR: pattern recognition receptor
Rantes: Ccl5
ROS: reactive oxygen species
Socs3: suppressor of cytokine signaling 3
SRs: scavenger receptors
TBI: traumatic brain injury
Tarc: Ccl17
T-bet: T-box–containing protein expressed in T cells
Tfam: mitochondrial transcription factor A
Tgfβ: transforming growth factor β
TH: tyrosine hydroxylase
Th1: T helper type 1
TLRs: Toll-like receptors
TMEV: Theiler's Murine Encephalomyelitis Virus
Tnfα: tumor necrosis factor α
Trem2: triggering receptor expressed on myeloid cells 2
VD: vascular dementia
Ym1: chitinase-3-like protein 3.
